# Development of a Core Outcome Set for Randomised Controlled Trials of Nursing Education: A Methodological Framework

**DOI:** 10.1155/2023/2107989

**Published:** 2023-03-29

**Authors:** Lingmin Chen, Shanxia Luo, Mutong Yang, Nian Li, Ying He, Yonggang Zhang

**Affiliations:** ^1^Department of Anesthesiology and National Clinical Research Center for Geriatrics, West China Hospital, Sichuan University and the Research Units of West China, Chinese Academy of Medical Sciences, Chengdu 610041, China; ^2^Mental Health Center, West China Hospital, Sichuan University, Chengdu 610041, China; ^3^West China Hospital of Stomatology, Sichuan University, Chengdu 610041, China; ^4^Department of Medical Administration, West China Hospital, Sichuan University, Chengdu 610041, China; ^5^Department of Integrated Traditional and Western Medicine, West China Hospital, Sichuan University, Chengdu 610041, China; ^6^Department of Periodical Press and National Clinical Research Center for Geriatrics, West China Hospital, Sichuan University, Chengdu 610041, China; ^7^Chinese Evidence-Based Medicine Center, West China Hospital, Sichuan University, Chengdu 610041, China; ^8^Nursing Key Laboratory of Sichuan Province, Chengdu 610041, China

## Abstract

**Background:**

Nursing educational research is very important for the development of the nursing discipline. There have been many randomised controlled trials (RCTs) of nursing education, and the outcomes are highly heterogeneous and waste resources. The study aims to report the methodological framework to establish a core outcome set (COS) for RCTs of nursing education.

**Methods:**

The study will be conducted in the following five steps: (a) establish nursing education COS working groups; (b) develop an initial list of outcomes of nursing education by systematic review and semistructured interview; (c) Delphi survey with different stakeholders to reach a preliminary consensus on the core outcome of nursing education; (d) expert consultation to form the outcome pool; (e) expert consensus meeting to form the nursing education COS.

**Results:**

The goal is to develop a COS that includes stakeholders' interest in nursing education to determine which outcomes should be reported and how they should be measured.

**Conclusions:**

By performing the study, the nursing education COS will be established, which will help to reduce reporting bias and resource waste, and provide enough results for nursing education systematic reviews.

## 1. Introduction

With the increasing aggravation of the ageing population, the nursing discipline's development is increasingly important [1, 2]. Nursing discipline depend on the development of nursing education [3–5]. New nursing teaching theories [6], teaching methods [7], and teaching models have been applied more and more in nursing education, such as the problem-based learning teaching model [8], integrated teaching model [9], core ability teaching model, massive open online course teaching model, and flipped classroom teaching model [10]. They have promoted the development of nursing education. In addition, new technologies such as artificial intelligence and virtual reality [11] have been increasingly applied to nursing education. With the introduction of new theories, methods, and models in nursing education, evaluating the effect of these methods is very important and designing randomised controlled trials (RCTs) is very important [12, 13].

In the nursing education field, an RCT is always performed to compare the different effects of the intervention and control methods and to identify the importance of the teaching method [14–17]. It aims to find the importance of improving outcomes and strategies for promoting nursing education [18–23]. There have been many RCTs on nursing education; however, the reported outcomes were heterogeneous [18–23]. Some used test scores [19], some used satisfaction [14], some used success rate [21], and some used ‘nursing students” knowledge and confidence [23]. Inconsistencies of outcomes will lead to the low use of studies in the systematic review [24]. It also makes the results inaccurate and unlikely to meet the needs of stakeholders. Therefore, outcomes must be standardised to reduce heterogeneity and waste resources for nursing education.

Developing a core outcome set (COS) will address these issues [25, 26]. The COS is a collection of the smallest and most important outcomes that should be measured and reported in a trial in the same health problem or social care area [27, 28]. It aims to improve research utility by involving stakeholders' perspectives and reducing inconsistency, reporting bias, and research waste [29–32]. In recent years, with the continuous application of new technologies in nursing education, implementing new RCTs requires the standardisation of outcomes. However, there was no COS for nursing education. Therefore, developing nursing education COS is needed. The development of COS will not only help to demonstrate different perspectives in this important area but also provide, for the first time, a standardised set of outcomes to be used in nursing education research and practice to assess the effect of nursing education and help to design research of nursing education.

## 2. Methods

### 2.1. Ethical Consideration and Registration

**Figure 1:**
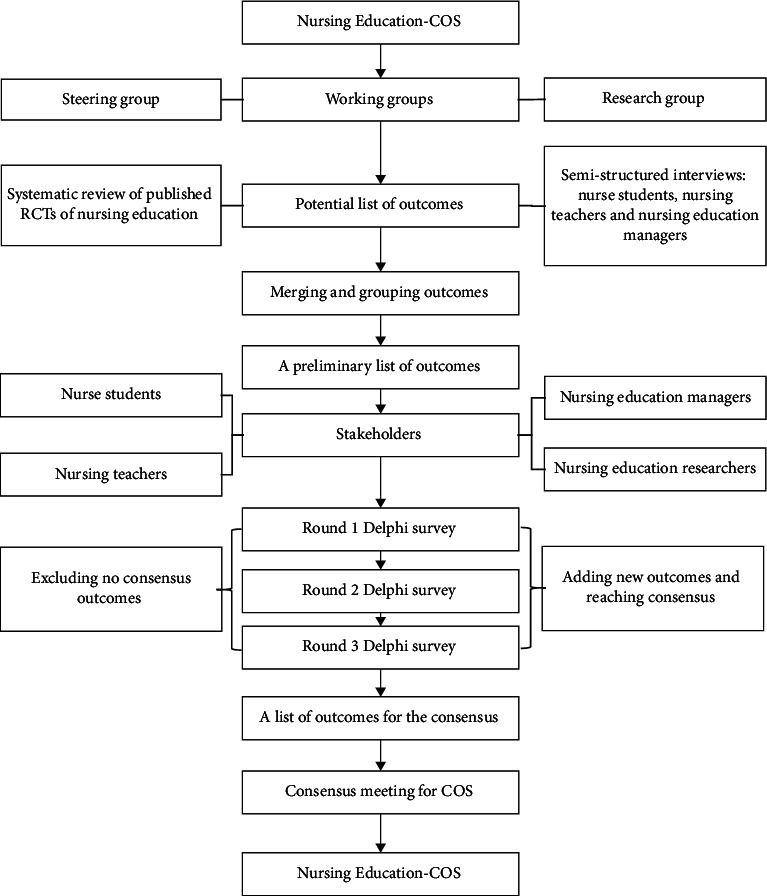
The development process for nursing education-COS.

The current study is a methodological framework for producing a COS but not for patients. When it is needed, ethical approval will be obtained from West China Hospital of Sichuan University. To be transparent, the methodological framework will be submitted to the peer-reviewed journal, and the methodological framework has also been submitted to register in the Core Outcome Measures in Effectiveness Trials (COMET) database. The methodological framework of the COS was reported according to the core outcome set standards for protocol items [33]. The main steps of the nursing education COS study are showed in Figure 1 and as follows.

### 2.2. Step 1: Establish Nursing Education COS Working Groups

According to the handbook, a steering group will be established [33]. The COS steering group will be composed of 3 multidisciplinary experts, including a nursing education manager with at least 20 years of work experience, an evidence-based medicine methodologist with at least ten years of research experience, and one nursing expert with at least 20 years of work experience. The primary responsibilities of the committee are: to determine the scope of COS, to approve COS methodological framework, to oversee the COS development process, and to provide advice and guidance for COS development as necessary. In addition, the research group will also be established. The research team will invite experts from universities in China, such as Sichuan University, Peking University, Fudan University, and so on.

### 2.3. Step 2: Develop an Initial Outcome List Covering All Relevant Outcomes

There will be three parts of the work, namely, [1] systematic review, [2] semistructured interview [24, 30, 34, 35], and merge and group outcomes.

#### 2.3.1. Systematic Review of the Outcomes in the RCTs for Nursing Education

*(1) Search Strategy*. The research group will search the following databases to identify outcomes reported in RCTs for nursing education published in 2021 and 2022: PubMed, Embase, The Cochrane Library, CNKI, CBM, and WanFang Data. The search strategies of PubMed are as follows: (nursing education OR nurse education OR nursing students OR nurse students OR nursing student OR nurse student) and (trial OR RCT OR randomised clinical trial OR systematic review OR meta-analysis). The language will be limited to English and Chinese.

*(2) Eligibility Criteria*. The inclusion criteria are as follows: the study should be RCT, the study should be for nursing education, the language should be Chinese or English, and the outcomes should be involved with the effectiveness of nursing education. The exclusion criteria are as follows: the page number of the study is only 1 page, the single author's publication, the study is for the patients, the study is for evaluating disease mechanism or pharmacokinetics of drugs, and the full text of the study could not be obtained.

*(3) Study Selection and Data Extraction*. Two authors will independently screen the titles and abstracts according to the inclusion and exclusion criteria to find the RCTs of nursing education. Any disagreement will be resolved by discussion [24, 30, 34, 35]. Two authors will independently extract the following items: the characteristics of the study, journal, author, and year; the characteristics of the intervention; and the outcomes. Any disagreements will be resolved by discussion. All outcomes will be included.

#### 2.3.2. Semistructured Interviews of Stakeholders

*(1) Participant Selection*. The outcomes generated by systematic reviews only reflect outcomes from research; the opinion of the nurse students, nursing teachers, and nursing education managers should also be considered. Thus, nurse students, nursing teachers, and nursing education managers will be invited to participate in the semistructured interviews. The inclusion criteria are as follows: (a) nursing students: undergraduate nursing students in school for at least one year, willing to participate in the study; (b) nursing teacher: at least five years of nursing education or clinical nursing work, had ever participated in nursing education research, bachelor's degree or above, willing to participate in the study; (c) nursing education manager: at least five years of nursing education managing work, had ever participated in nursing education research, bachelor's degree or above, willing to participate in the study.

*(2) Recruitment and Data Collection*. The authors will invite nurse students, nursing teachers, and nursing education managers from the West China Hospital of Sichuan University. The author will also invite nurse students, nursing teachers, and nursing education managers from other nursing schools to participate. As shown in previous studies [24, 30, 34, 35], when the sample size is 30, it will achieve data saturation; therefore, the author will include 30 nurse students, 30 nursing teachers, and 30 nursing education managers. If new information is generated in the interview, the sample size will be expanded as previously suggested [24, 30, 34, 35].

The authors, who have been trained in qualitative research methods, will conduct the interview and introduce the purpose and content of the interview to the participants. All participants will receive and read separate written information. The participants who agree to participate in the interview will provide their signed informed consent. After obtaining the participants' consent, the interview's content will be recorded in audio. The information on demographic characteristics will also be recorded. The interview will be mainly performed in China. Face-to-face interviews or online interviews will be used when it is possible.

The outline of the semistructured interviews for nursing teachers and nursing education managers: How long have you been a nursing teacher or a nursing education manager? What intervention will you give to nursing education? Which outcome do you think is the best intervention to improve nursing education? Please write down at most five outcomes that you think are important for nursing education.

The outline of the semistructured interviews for nursing students is as follows: What is your grade? What intervention have you received because of nursing education from your nursing teacher? Which outcome of intervention do you want to improve after education? Which outcome do you want the intervention to improve most?.

*(3) Data Analysis*. Two authors will independently analyse the results of semistructured interviews to identify the important outcomes for RCTs of nursing education. The authors will conduct all interview transcriptions using the qualitative analysis software NVivo 12 plus. Two researchers will first read all transcripts to familiarise themselves with the data and develop a structured coding tree that starts with an inductive open coding. The transcripts and open coding will be initially coded individually by the two researchers. To ensure consistency and reliability of the process, themes will be sought, reviewed, defined, and named. Inconsistency will be discussed until reaching a consensus. After review by the steering committee, the new outcomes will be added to the list of outcomes.

#### 2.3.3. Merging Outcomes and Grouping Outcomes into Different Domains

Based on previous COS studies [24, 30, 34, 35], the outcomes from the systematic review and the semistructured interviews will be merged and grouped into different domains. Nursing student-reported, teacher-reported, and nursing education manager-reported outcomes will be categorised [24, 30, 34, 35]. Two researchers will independently conduct this process. Any discrepancies will be resolved through discussion or by consulting the steering group.

### 2.4. Step 3: Delphi Survey with Different Stakeholder Groups to Prioritise the Outcomes

Delphi survey will be performed as previously reported [24, 30, 34, 35]. The Delphi manager is based on a web system to build and administer the Delphi surveys [24, 30, 34, 35].

#### 2.4.1. Stakeholder Selection

The researchers will invite nursing teachers, nursing students, nursing educational managers, and nursing education researchers to participate in the three rounds of the Delphi survey [24, 30, 34, 35]. Representatives of the stakeholder groups are as follows: (a) nursing students: undergraduate nursing students in school for at least one year who are willing to participate in the study; (b) nursing teacher: at least five years of nursing education or clinical nursing work, had ever participated in nursing education research, bachelor's degree or above, and willing to participate in the study; (c) nursing education manager: at least five years of nursing education managing work, had ever participated in nursing education research, bachelor's degree or above, and willing to participate in the study; and (d) nursing education researcher: first author or contact author of publication of nursing education, at least five years of nursing education or nursing clinical research work, bachelor's degree or above, and willing to participate in the study. There is no restriction on the geographical area of participants. As there has been no standard sample size calculation method in the Delphi survey in the development of the COS study [24, 30, 34, 35], referring to the previous COS studies, the researchers plan to select 30 participants for each stakeholder group.

#### 2.4.2. Consensus Standards

The consensus standards will be defined as previously reported: (a) ≥70% of participants scored the outcomes as 7 to 9, and <15% of the participants scored the outcomes as 1 to 3, and the outcomes will be included; (b) ≤50% participants scored the outcome as 7–9, and the outcomes will be excluded [24, 30, 34, 35].

#### 2.4.3. Three Rounds of Delphi Survey and Data Analysis

All candidate outcomes of nursing education will be included in the questionnaire [24, 30, 34, 35]. The questionnaire will be in two versions: for nursing teachers, nursing education managers, and researchers, and for nursing students. All participants will register in the Delphi manager and sign the informed consent [24, 30, 34, 35]. All participants will score the candidate items via the online survey and can add new outcomes that are not included in the outcome list [24, 30, 34, 35]. Additional materials will also be sent to them for reference. They can also contact the authors for further details if they have any questions. Each round of the Delphi survey will be planned in three weeks, and the author will send two emails at the end of the first and second weeks to remind the participants. If the response rate is <80% at the end of the third week, the time will be prolonged.

Descriptive analysis will be applied to analyse the results of the Delphi survey of different stakeholders. An outcome is scored as ≥4 by ≥70% of the participants in any stakeholder group who complete the questionnaire in round 1 will be included in round 2. In round 2, the participants will need to re-score the outcomes, the outcomes that agree with the consensus standards will be directly included in the consensus meeting, and the rest of the outcomes will be surveyed in round 3.

### 2.5. Step 4: Expert Consultation and form the Outcome Pool for the Expert Consensus Meeting

Expert consultation will be performed after analysing the results of the Delphi survey. The research team will invite one nursing teacher, one nursing education manager, one nursing education researcher to work with the steering committee to discuss the results of Delphi and determine the outcome pool for an expert consensus meeting.

### 2.6. Step 5: Consensus Meeting

After the development of the outcome pool, a face-to-face consensus meeting will be held with key stakeholders to finalise the COS. The consensus meeting will be held in Chengdu in China.

#### 2.6.1. Participants of the Consensus Meeting

As reported in previous studies, there is no standard method to calculate the sample size for the consensus meeting in the development of COS studies [30]. Therefore, the researchers will invite 20 to 25 stakeholders to participate in the consensus meeting, including (a) nursing teachers who have more than ten years of teaching experience; (b) nursing education managers who have more than ten years of nursing education managing work; (c) evidence-based medicine methodologists; (d) nursing education researchers with a master's degree or above; (e) journal editors; and (f) nursing students.

#### 2.6.2. Process of the Consensus Meeting

First, the potential outcomes from the survey will be reported to the stakeholders, and the stakeholders will decide whether the outcomes meet the consensus criteria [24, 30, 34, 35]. In addition, the less consistent outcomes will be discussed [24, 30, 34, 35]. Finally, the stakeholders will vote for each outcome. The detailed information of the consensus definition will be based on the previously mentioned [24, 30, 34, 35]. The nursing education COS will finally be formed.

## 3. Discussion

Nursing education research is important for developing nursing discipline and has significance for nursing management [4, 5]. However, the results have been very heterogeneous due to the heterogeneous outcomes of previous RCTs in nursing education. Many results cannot be included in a systematic review to produce evidence. Therefore, it is necessary to carry out the study of COS for nursing education.

At present, COS studies have been published in several disciplines, promoting the standardisation of outcomes in trials and reducing research waste [24, 30, 34, 35]. However, there is a need for COS in nursing education. Due to the wide range of nursing disciplines, the COS in nursing education will promote nursing education to a certain extent and improve the results of nursing education.

The current study reports a comprehensive methodological framework for COS of RCTs of nursing education by conducting a systematic review, semistructured interview, Delphi survey, expert consultation, and consensus meeting [24], which can ensure the feasibility and promotion of COS in future RCTs for nursing education. The development of COS will help the consistency of reporting study outcomes of RCTs for nursing education and reduce reporting bias [30]; then, the results of different RCTs can be compared and merged to improve the value of interventions and reduce waste resources for nursing education.

## 4. Implications for Nursing Management

Standardisation of the outcomes and establishing a COS will be helpful to reduce reporting bias, reduce resource waste, and help the management of nursing education research. The current methodological framework will be helpful to develop a COS for RCTs in nursing education; thus, it will help the management of nursing research and nursing education.

## Data Availability

No underlying data was collected or produced in this study.
